# Comparison of the efficacy and safety of URSL, RPLU, and MPCNL for treatment of large upper impacted ureteral stones: a randomized controlled trial

**DOI:** 10.1186/s12894-017-0236-0

**Published:** 2017-06-29

**Authors:** Yunyan Wang, Bing Zhong, Xiaosong Yang, Gongcheng Wang, Peijin Hou, Junsong Meng

**Affiliations:** Department of Urology, Huai’an First People’s Hospital, Nanjing Medical University, No. 6 West Beijing Road, Huai’an, Jiangsu 223300 China

**Keywords:** Ureteral calculi, Ureteroscopy, Nephrostomy, Percutaneous, Laparoscopy

## Abstract

**Background:**

There are three minimally invasive methods for the management of large upper impacted ureteral stones: mini-percutaneous nephrolithotomy (MPCNL), transurethral ureteroscope lithotripsy (URSL), and retroperitoneal laparoscopic ureterolithotomy (RPLU). This study aimed to compare MPCNL, URSL, and RPLU, and to evaluate which one is the best choice for large upper impacted ureteral stones.

**Methods:**

Between January 2012 and December 2015, at the Department of Urology, Huai’an First People’s Hospital, 150 consecutively enrolled patients with a large upper impacted ureteral stone (>15 mm) were included. The patients were randomly divided (1:1:1) into the MPCNL, URSL, and RPLU groups. The primary endpoint was success of stone removal measured 1 month postoperatively and the secondary endpoints were intraoperative and postoperative parameters and complications.

**Results:**

Fifteen patients needed auxiliary ESWL after URSL, and 3 patients after MPCNL, but none after RPLU. The stone clearance rate was 96% (48/50) in the MPCNL group and 72% (33/46) in the URSL group. In the RPLU group the stones were completely removed and the stone clearance rate was 100% (48/48) (*P* = 0.021 vs. URSL; *P* = 0.083 vs. MPCNL). Operation-related complications were similar among the three groups (all *P* > 0.05). Hospital stay was shorter in the URSL group compared with MPCNL (*P* = 0.003). Operation time was the shortest with URSL and the longest with MPCNL (all *P* < 0.05).

**Conclusions:**

MPCNL and RPUL are more suitable for upper ureteral impacted stones of >15 mm. URSL could be considered if the patient is not suitable for general anesthesia, or the patient requests transurethral uretroscopic surgery.

**Trial registration:**

This study was registered with the Chinese Clinical Trial Registry (Registration number: ChiCTR-INR-17011507; Registration date: 2017–5-22).

## Background

Urinary lithiasis, where stones known as calculi form in the urinary system, is a common problem for more than 12% of the population [1], that is increasingly prevalent in many populations [2–4]. The definition of an impacted ureteral stone is one that stays in the same location at least for 2 months and results in ureteral obstruction [5]. Such stones can cause pain and lead to hydronephrosis or urinary tract infections, which may result in loss of renal function [6]. Generally, the transverse diameter of an impacted ureteral stone is longer than the ureter caliber. Other characteristics such as a large volume, anomalous shape, and uneven density, will result in ureteral obstruction, nephrohydrosis, and pyonephrosis. Secondary infection and the immune response to foreign material resulting from chronic oppression, pathological lesions such as ureteral polyps, and stricture also occur in the stone site [7]. Therefore, these stones require interventions for their removal. Various treatment modalities are available, from open ureterolithotomy to modern endourologic procedures [8].

Before the 1980s, the majority of large upper ureteral stones required open operation for their removal [9]. With the development of minimally invasive techniques, various treatment options have become available such as extracorporeal shock wave lithotripsy (ESWL), ureteroscopic lithotripsy (URSL), percutaneous nephrolithotomy (PCNL), as well as retroperitoneal ureterolithotomy (RPUL), all with different efficacy rates [10].

In most cases, ESWL is the first line choice for upper ureteral stones that do not pass spontaneously, but for large ureteral impacted stones, ESWL has been less successful [11]. Therefore, the debate over the optimal treatment for larger stones of 15 mm diameter or more remains [8]. When the stones are located in a high position and are close to the renal pelvis there is a risk of the stones returning to the pelvis, which results in the failure of URSL [12]. Both PCNL and mini-PCNL (MPCNL) have been used more often to treat upper ureteral stones in recent years [13]. With the improvement of laparoscopic techniques and equipment, retroperitoneoscopic ureterolithotomy (RPUL) has also become a popular choice [6].

All these mini-invasive treatment approaches can be used to treat impacted upper ureteral stones, but how to select one and what is their efficiency remains controversial. A meta-analysis by Torricelli et al. [14] showed that the outcomes of RPUL were more favorable than for semi-rigid ureteroscopic lithotripsy, making it the treatment of choice when flexible ureteroscopy is not available. PCNL has been reported to have the same efficacy as laparoscopic pyelolithotomy, but to be associated with better operative parameters [15]. Therefore, the aim of this study was to compare three minimally invasive methods; URSL, MPCNL and RPUL to evaluate which one is the best choice for large upper ureteral stones (>15 mm) in terms of efficacy and safety.

## Methods

### Clinical materials

From January 2012 to December 2015, 150 consecutive patients with upper ureteral stones who were referred to the department of Urology, Huai’an First People’s Hospital (Huai’an, Jiangsu Province) were included in the study.

The inclusion criteria were patients with a single upper ureteral stone (located below the ureteropelvic junction to the superior aspect of sacroiliac joint); the stone was >15 mm along its longest diameter as revealed by kidney-ureter-bladder (KUB) abdominal plain film. The exclusion criteria were those patients with a history of any intervention operation on the corresponding ureter, radiolucent stones, active infection, or urinary tract abnormalities, coagulopathy, or pregnancy, as well as those patients requiring simultaneous treatment of a kidney stone. The patients all agreed to enter the study, and this study was approved by the Ethic Committee of Huai’an First People’s Hospital, Nanjing Medical University (IRB-PJ2012–015-01). A written informed consent was obtained from all subjects prior to the start of the trial.

In addition to routine history and clinical examinations, the investigations included assessment of the hemoglobin and serum creatinine values, full coagulation profile, ultrasonography, and KUB plain film. Excretory urography was performed if the serum creatinine was normal. Urine specimens were obtained for culture. A sensitive antibiotic was given to the patients with positive cultures to control the infection before surgical intervention.

The patients included in the study were randomly divided (1:1:1) into three groups by use of a computer generated random number table.

### Procedures

All procedures were performed by the same physician.

#### URSL

The patient was under spinal or general anesthesia and placed in the lithotomy position. An 8 to 9.8 F rigid ureteroscope (Richard Wolf GmbH, Knittlingen, Germany) was used for uteroscopy and access was provided by retrograde insertion of a 0.038-in. floppy tip guide wire over which the ureteroscope was introduced into the ureter without dilating the ureteral orifice. The stones were fragmented with a holmium YAG laser through the ureteroscope. A double-J stent was placed in cases with large residual stones, significant mucosal edema, stone impaction, or probable ureteral trauma. The stent was removed when the patient was stone-free on follow-up evaluation as an outpatient.

#### MPCNL

Under general anesthesia, the patient was placed in the lithotomy position and an external 5 Fr or 6 Fr ureteral catheter was inserted to the target ureter under direct ureteroscopic vision. Then the patient was rotated to the prone position with a pack under the ipsilateral hemi-pelvis. An ultrasound-guided percutaneous puncture was made by the urologist with an 18-gauge puncture needle being pushed into the designated calyx. A flexible guide wire was then inserted through the calyceal puncture into the renal pelvis and across the ureteropelvic junction into the ureter. An 8 Fr fasical dilator was employed initially, and the caliber was increased gradually by progressive 2 Fr fascial dilators along the guide wire, until the percutaneous nephrostomy tract was dilated to 18 Fr. A matched peel-away sheath was inserted into the renal collecting system. All the stones were fragmented with a Swiss lithoclast used as the sole device for using a 2.4 F (0.8-mm thick), 668-mm-long probe and stone debris were flushed out by a water flow produced by an endoscopic perfusion pump (EMS - Electro medical Systems S.A., Nyon, Switzerland). At the end of the procedure, a 5 Fr double-J stent was indwelled via the percutaneous access with the assistance of the guide wire. All the percutaneous tracts were inserted with a 16 Fr silastic nephrostomy tube.

#### RPLU

Under general endotracheal anesthesia, the patients were placed in the lateral decubitus position. A skin incision was made at the tip of the 12th rib and the aponeurosis was bluntly perforated under safe control of both hands. A retroperitoneal working space was created with a self-made expansion balloon that was inserted by pushing the peritoneum forward. Approximately 800 ml of sterile saline solution was injected into the dissection balloon through the transparent channel. The retroperitoneal space was bluntly dissected and the dissection balloon was removed. A 5- or 10-mm trocar was then inserted under the subcostal margin in the anterior axillary line. A 10-mm trocar was also placed above the iliac crest in the midaxillary line and this space was filled with CO_2_ pneumoretroperitoneum for the laparoscope (Karl Storz Endoskope, Tuttlingen, Germany). Within the retroperitoneal space the psoas muscle and other important landmarks were easily recognized. The Gerota’s fascia was incised parallel to the psoas muscle. Renal vessels were clearly visible as pulsing. Extraperitoneal adipose tissue was removed and the ureter was recognized on the psoas muscle. The stone location could be identified by a conspicuous bulge as the ureter was dissected. The ureteral wall was longitudinally incised by a cold knife over the bulge and the stone was extracted and removed through the first port. An indwelling double-J ureteral stent was placed through the incision. Intracorporeal suturing was used to close the ureteral incisions with 4–0 absorbable sutures.

### Appraisal methods

Radiologists were blind to patient data during all follow-up examinations. All the patients accepted the KUB plain film examination within 3 days of their procedure. ESWL on residual stone was performed 1 week after surgery in the URSL group, and 2 weeks after surgery in the MPCNL group. For these patients, KUB plain film examination was performed again within 3 days after their procedure.

The primary outcome was whether treatment was successful. Successful treatment was defined as complete removal of the target stones or the presence of peripheral small insignificant gravel (<4 mm in diameter) [16]. According to the Chinese guidelines of medicine, stones of <4 mm are considered to be able to pass by themselves. Therefore, obtaining fragments <4 mm was considered successful [16]. If the residual stone diameter was >4 mm, then auxiliary ESWL treatment was undertaken.

One month after surgery, the patient returned to the hospital to remove the double-J stent and to be reexamined by KUB film. Stone clearance was defined as the absence of stone debris on the KUB film, and the stone clearance rate was calculated.

The secondary outcomes were intraoperative and postoperative parameters and complications. Complications arising intraoperatively and postoperatively, and hospitalization days after surgery were assessed. The Clavien method was used for the classification of surgical complications [17]. The patients were followed up at 6 and 12 months to ensure that there was no novel stone or stenosis.

### Statistical analysis

No power calculation was performed before beginning the trial and the sample size was based on convenience. Nevertheless, a post hoc power analysis based on the primary outcome revealed that our experiment had a 95% power to detect the differences in the primary outcome with a two-tailed α = 0.05. SPSS 16.0 (IBM Corp., Armonk, NY, USA) was used for statistical analysis. The continuous or categorical data are presented as mean ± standard deviation (SD), frequency, percentile, and range, as appropriate. For normally distributed continuous variables, analysis of variance (ANOVA) was used to detect differences among the groups and the Tukey’s post hoc test was used. Variables in the contingency table were analyzed by the χ^2^ test (or the Fisher exact test). *P* < 0.05 indicated statistical significance.

## Results

### Baseline data

**Fig. 1:**
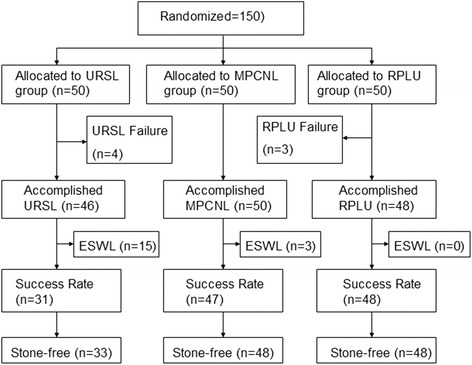
Patient flowchart

There were 88 men and 62 women. None of the patients withdrew from the study (Fig. 1). The detailed characteristics of the patients are presented in Table 1 and Fig. 1 shows the patient flowchart. There were no statistically significant differences among the three groups for stone size and nephrohydrosis extent (both *P* > 0.05; Table 1). All patients were followed up at 6 and 12 months.

**Table 1:** Baseline characteristics of the included patients

Variable	URSL group	MPCNL group	RPUL group	*P* value^a^	*P* value^b^	*P* value^c^
	*N* = 50	*N* = 50	*N* = 50			
Mean age (years)	42 ± 14	41 ± 15	44 ± 11	0.769	0.385	0.581
Male/female	28/22	31/19	29/21	0.274	0.162	0.469
Side (left / right)	26/24	27/23	29/21	0.481	0.376	0.583
Mean stone size (mm)	16.8 ± 2.1	19.3 ± 1.8	18.8 ± 1.4	0.677	0.943	0.876
Hydronephrosis (mm)	35.8 ± 5.5	40.2 ± 7.8	38.4 ± 6.9	0.264	0.573	0.815

^a^URSL vs. MPCNL; ^b^URSL vs. RPUL; ^c^MPCNL vs. RPUL

All procedures in the MPCNL group were completed at the first attempt. Four patients failed to undergo the designated procedure in the URSL group because the ureteroscope could not approach the stone location. One of these patients then underwent URSL successfully 5 days after placing the double-J stent. The other three patients underwent open surgery to remove the stone. Two patients in the RPLU group failed to undergo the procedure because the stone returned to the renal pelvis and the stone was removed by open surgery. These six cases of failure to perform the procedure at the first attempt were not included in the statistics data of stone clearance rate. ESWL on the residual stone was performed 1 week after surgery in URSL group (*n* = 15), and 2 weeks after surgery in MPCNL group (*n* = 3).

### Primary endpoint

The successful treatment rate was 31/50 (62%) in the URSL group, 47/50 (94%) in the MPCNL group, and 48/50 (96%) in the RPUL group. The differences were not significant among the three groups (Table 2), but differences of stone clearance rate 1 month after operation among the three groups were statistically significant (*P* < 0.05). Auxiliary ESWL was required in a large number of patients in the URSL group (*n* = 15), but only in three patients in the MPCNL group and in none in the RPUL group.

**Table 2:** Patient outcomes after the procedure

Variable	URSL group	MPCNL group	RPUL group	*P* value^a^	*P* value^b^	*P* value^c^
Success rate	31/50 (62%)	47/50 (94%)	48/50 (96%)	<0.001	<0.001	0.698
Mean operation time (min)	55.7 ± 23.9	125.6 ± 41.2	99.5 ± 34.6	<0.001	0.027	0.012
Hospital stay after surgery (d)	2.5 ± 1.3	6.8 ± 2.6	4.3 ± 2.2	0.003	0.056	0.063
Auxiliary ESWL after 3 days	15/46 (32.6%)	3/50 (6%)	0/48 (0%)	<0.001	<0.001	<0.001
Stone-free rate after 1 month	33/46 (72%)	48/50 (96%)	48/48 (100%)	0.035	0.021	0.083

^a^URSL vs. MPCNL; ^b^ URSL vs. RPUL; ^c^ MPCNL vs. RPUL

### Secondary endpoints

There were no statistically significant differences in the length of major axis and surface area of stones as well as in the complications and morbidity (*P* > 0.05). The mean operation time was significantly different among the groups; the shortest was in the URSL group at 55.7 ± 23.9 min and the longest was in the MPCNL group at 125.6 ± 41.2 min (*P* < 0.05). A similar result was found with the length of hospital stay: a significantly shorter time was needed after URSL (2.5 ± 1.3 days) than after RPUL (4.3 ± 2.2 days) and the longest hospital stay was after MPCNL (6.8 ± 2.6 days, all *P* < 0.05).

### Adverse effects or complications

There were no severe complications in any of the patients. In the URSL group, the main postoperative complications were stone fragment migration, perforation, and ureteral stricture. In the MPCNL group, bleeding occurred in five cases and three of them needed a blood transfusion. Three cases had fever because of urosepsis. In the RPUL group, six complications occurred, including abdominal distention caused by peritoneal rupture, subcutaneous emphysema, and urine leakage (Table 3).

**Table 3:** Complications and adverse events

Variable	URSL group	MPCNL group	RPUL group	*P* value^a^	*P* value^b^	*P* value^c^
Grade I						
Pain	6/46(13%)	8/50(16%)	9/48(18%)	0.276	0.027	0.795
Fever	2/46(4.3%)	3/50(6%)	2/48(4.2%)	0.735	0.658	0.743
Nausea/vomiting	2/46(4.3%)	1/50(2%)	3/48(6%)	0.273	0.342	0.042
Urine leakage	0/46(0%)	0/50(0%)	3/48(6%)	NS	<0.001	<0.001
Grade II						
Minor pelvic/ureter perforation	3/46(6.5%)	0/50(0%)	0/48(0%)	<0.001	<0.001	NS
Urinary tract infection	1/46(3%)	1/50(2%)	0/48(0%)	NS	<0.001	<0.001
Ureteral stricture	2/46(4.3%)	0/50(0%)	0/48(0%)	<0.001	<0.001	NS
Grade III						
Blood transfusion	0/46(0%)	3/50(6%)	0/48(0%)	<0.001	NS	<0.001
Grade III - V	0/46(0%)	0/50(0%)	0/48(0%)	NS	NS	NS

^a^URSL vs. MPCNL; ^b^URSL vs. RPUL; ^c^ MPCNL vs. RPUL; *NS* No Significance

## Discussions

There are many treatments for impacted upper ureteral stones, including URSL, MPCNL, and RPLU. Because impacted stones usually are wrapped around or adhere to an ureteral polyp, ESWL is often not effective [18]. Indeed, White et al. reported that if upper ureteral stone diameter was smaller than 10 mm, stone clearance rate by ESWL was 69%, however; when the diameter was larger than 10 mm, it was 59% [18]. It was also reported that when upper ureteral stones are larger than 10 mm, stone clearance rate by ESWL was only 42% [19].

Each method has its pros and cons. Indeed, RPUL takes a long time, but has more chance of success and a lower requirement for ESWL; it also results in fewer complications, but the surgeons have to be adept at local anatomy [10]. PCNL has a good efficacy, but may result in large surgical trauma and bleeding, complicating the recovery of the patients and prolonging hospitalization [10, 20]. URSL is not as effective as RPUL and PCNL, and is prone to move the calculi upward; nevertheless, the surgical trauma by URSL is minimal, leading to short recovery [10, 21]. A meta-analysis by Torricelli et al. [14] showed that the outcomes of RPUL were more favorable than for semi-rigid ureteroscopic lithotripsy, making it the treatment of choice when flexible ureteroscopy is not available.

Ureteroscopic surgery is a minimally invasive procedure, which has a good acceptance for patients and the patients restore quickly after operation. In this study the success rate was 62% and the stone clearance rate was 72% 1 month after operation in the URSL group. The success rate was previously reported to be 35–87% by URSL [22, 23]. Usually, general anesthesia is required in MPCNL and RPLU, while URSL can be performed under spinal anesthesia. So, URSL is especially appropriate for patients who are not suitable for general anesthesia.

However, there are several disadvantages with URSL when dealing with impacted upper ureteral stones. Firstly, the stone clearance rate is relatively low. In most cases, the stones are large and near to renal pelvis. During URSL, the stone and its debris are inclined to return to the renal pelvis under the flushing fluid, resulting in residual stones. Secondly, ESWL is often needed as auxiliary treatment after surgery. Chen et al. [24] reported that ESWL as an auxiliary procedure was 16%. In our study, as an auxiliary procedure, the ESWL treatment rate was 32.6%.

In this study, there were two cases of ureteral stricture postoperatively in the URSL group, which may correlate with long-term obstruction, chronic inflammation and polyp proliferation. Moreover, the holmium laser crushed the stone at an identical spot during the operation time, which would aggravate the ureter mucosal membrane damage, inevitably resulting in occurrence of ureteral stricture. For these patients, we suggest that the double-J stent indwelling time should be increased to 8–12 weeks. Regarding the obvious polyp proliferation cases, urotroscopy was required to detect ureteral stricture when the double-J stent was removed.

With the improvement of endoscopy and lithotripsy instruments in the last decade, PCNL, instead of open surgery, has already become an option for minimally invasive lithotripsy for kidney stones and is gradually being adopted for upper ureteral stones [11, 25]. Karami [26] and colleagues compared URSL and PCNL in 70 cases of upper ureteral impacted stones >1 cm. The results showed that the stone clearance rate was 96% in the PCNL group, while the stones of 32% patients in the URSL group returned to the renal pelvis and needed ESWL after surgery. The authors thought that PCNL was the first choice for these kinds of stones. A similar conclusion was drawn in another study of 53 patients who underwent either PCNL or URSL. The stone-free rate at 1-month follow-up was 95.4% in the PCNL group and 58% in the URSL group, and eight patients had upward migrating stones during the URSL procedure; they were treated by ESWL [27]. Out results show that the stone clearance rate was 96% 1 month after surgery in the MPCNL group. We found similar results when comparing URSL and MPCNL, but the complications in the groups were similar. In our opinion, intrapoerative puncture is not difficult for cases of moderate or severe hydronephrosis resulting from upper ureteral impacted stones.

RPLU was first reported by Gaur [28] in 1994. As we know, RPLU has many merits, such as high stone-free rate, less blood loss, less incision pain, and shorter hospitalization time [29]. Therefore, RPLU should be considered for safe and effective treatment for reducing ureteral obstruction in selected patients with large proximal ureteric stones [6, 15, 30, 31]. In this study, the stone-free rate was 100% 3 days after operation in the RPLU group.

We realized that RPLU should be selected for upper ureteral stones when they are combined with mild hydronephrosis, when the ureteropelvic junction is angled, or when it is difficult for PCNL to arrive at the stone position. If the stone is near to the UPJ and hydronephrosis is obvious, the possibility of stones going back into the renal pelvis during the operation increases greatly, which will affect the success rate of the RPLU procedure. In this study, there was no ureteral stricture after RPLU during the long-term follow-up, which might contribute to ureter incision going along the ureteral axis and little heat damage of the ureteral mucosal membrane. However, impacted stones might adhere to the ureteral wall so closely that it is difficult to identify the ureter and remove the stone using RPLU [27]. Therefore, RPLU should only be conducted by urologists who have mastered the subtle skills needed for the laparoscopic technique.

This study has some limitations. The sample was from one single center. Although it was larger than many studies, it remains quite small. Studies from multiple centers would provide more weight to these results. There was no postoperative CT examination 1 month after the operation when the stone clearance rate was calculated. The follow-up of 6–12 months was quite short, so we cannot provide any comparison of recurrence rates or long term complications between the groups.

## Conclusions

In our opinion, MPCNL and RPUL are more suitable for upper ureteral impacted stones with a diameter of >15 mm. URSL could be considered if the patient is not suitable for general anesthesia, or the patient requests transurethral uretroscopic surgery.

## Abbreviations

CTU: computed tomography urography
ESWL: Extracorporeal shock wave lithotripsy
KUB: kidney-ureter-bladder
MPCNL: mini-percutaneous nephrolithotomy
PCNL: percutaneous nephrolithotomy
RPLU: retroperitoneal laparoscopic ureterolithotomy
SD: standard deviation
UPJ: ureteropelvic junction
URSL: ureteroscope lithotripsy
